# Burnout among primary health-care professionals in low- and middle-income countries: systematic review and meta-analysis

**DOI:** 10.2471/BLT.22.288300

**Published:** 2022-04-29

**Authors:** Tanya Wright, Faraz Mughal, Opeyemi O Babatunde, Lisa Dikomitis, Christian D Mallen, Toby Helliwell

**Affiliations:** a School of Medicine, David Weatherall Building, Keele University, University Road, Keele, Staffordshire, ST5 5BG, England.; b Kent and Medway Medical School, University of Kent and Canterbury Christ Church University, Canterbury, England.

## Abstract

**Objective:**

To estimate the prevalence of burnout among primary health-care professionals in low- and middle-income countries and to identify factors associated with burnout.

**Methods:**

We systematically searched nine databases up to February 2022 to identify studies investigating burnout in primary health-care professionals in low- and middle-income countries. There were no language limitations and we included observational studies. Two independent reviewers completed screening, study selection, data extraction and quality appraisal. Random-effects meta-analysis was used to estimate overall burnout prevalence as assessed using the Maslach Burnout Inventory subscales of emotional exhaustion, depersonalization and personal accomplishment. We narratively report factors associated with burnout.

**Findings:**

The search returned 1568 articles. After selection, 60 studies from 20 countries were included in the narrative review and 31 were included in the meta-analysis. Three studies collected data during the coronavirus disease 2019 pandemic but provided limited evidence on the impact of the disease on burnout. The overall single-point prevalence of burnout ranged from 2.5% to 87.9% (43 studies). In the meta-analysis (31 studies), the pooled prevalence of a high level of emotional exhaustion was 28.1% (95% confidence interval, CI: 21.5–33.5), a high level of depersonalization was 16.4% (95% CI: 10.1–22.9) and a high level of reduced personal accomplishment was 31.9% (95% CI: 21.7–39.1).

**Conclusion:**

The substantial prevalence of burnout among primary health-care professionals in low- and middle-income countries has implications for patient safety, care quality and workforce planning. Further cross-sectional studies are needed to help identify evidence-based solutions, particularly in Africa and South-East Asia.

## Résumé

**Objectif:**

Estimer la prévalence de l’épuisement professionnel (burnout) chez les professionnels des soins de santé primaires dans les pays à revenu faible ou intermédiaire et identifier les facteurs associés à l’épuisement professionnel.

**Méthodes:**

Nous avons effectué une recherche systématique dans neuf bases de données jusqu’en février 2022 afin d’identifier les études portant sur l’épuisement chez les professionnels des soins de santé primaires dans les pays à revenu faible ou intermédiaire. Il n’y avait aucune limitation linguistique et nous avons inclus des études observationnelles. Deux examinateurs indépendants ont effectué le screening, la sélection des études, l’extraction des données et l’évaluation de la qualité. Une méta-analyse à effets aléatoires a été utilisée pour estimer la prévalence globale de l’épuisement professionnel, évaluée à l’aide des sous-échelles d’épuisement émotionnel, de dépersonnalisation et d’accomplissement personnel du Maslach Burnout Inventory. Voici un rapport narratif des facteurs associés à l’épuisement professionnel.

**Résultats:**

Nos recherches ont permis d’identifier 1568 articles. Après sélection, 60 études provenant de 20 pays ont été incluses dans l’examen narratif, et 31 ont été incluses dans la méta-analyse. Trois études ont recueilli des données pendant la pandémie de Covid-19, mais ont fourni des preuves limitées de l’impact de cette maladie sur l’épuisement professionnel. La prévalence globale à point unique de l’épuisement professionnel variait de 2,5 à 87,9% (43 études). Dans la méta-analyse (31 études), la prévalence collective d’un niveau élevé d’épuisement émotionnel était de 28,1% (intervalle de confiance (IC) à 95%: 21,5–33,5), un niveau élevé de dépersonnalisation était de 16,4% (IC à 95%: 10,1–22,9) et un niveau élevé d’accomplissement personnel réduit était de 31,9% (IC à 95%: 21,7–39,1).

**Conclusion:**

La prévalence notable de l’épuisement professionnel chez les professionnels des soins de santé primaires dans les pays à revenu faible ou intermédiaire a des implications pour la sécurité des patients, la qualité des soins et la planification des effectifs. D’autres études transversales sont nécessaires pour aider à identifier des solutions empiriques, en particulier en Afrique et en Asie du Sud-Est.

## Resumen

**Objetivo:**

Estimar la prevalencia del desgaste profesional entre los profesionales de la atención primaria de salud en los países de ingresos bajos y medios e identificar los factores que se asocian a este síndrome.

**Métodos:**

Se realizaron búsquedas sistemáticas en nueve bases de datos hasta febrero de 2022 para identificar estudios que investigaran el desgaste profesional en profesionales de la atención primaria de países de ingresos bajos y medios. No hubo limitaciones de idioma y se incluyeron estudios observacionales. Dos revisores independientes completaron el cribado, la selección de estudios, la extracción de datos y la evaluación de la calidad. Se utilizó el metanálisis de efectos aleatorios para estimar la prevalencia global del desgaste profesional, evaluada mediante las subescalas de agotamiento emocional, despersonalización y realización personal del Maslach Burnout Inventory. En este documento, se describen los factores asociados al síndrome de desgaste profesional.

**Resultados:**

La búsqueda arrojó 1568 artículos. Tras la selección, se incluyeron 60 estudios de 20 países en la revisión narrativa y 31 en el metanálisis. Tres estudios recopilaron datos durante la pandemia de la enfermedad por coronavirus de 2019, pero proporcionaron pruebas limitadas sobre el impacto de la enfermedad en el desgaste profesional. La prevalencia global de un único punto sobre el desgaste profesional osciló entre el 2,5 y el 87,9 % (43 estudios). En el metanálisis (31 estudios), la prevalencia conjunta de un nivel alto de agotamiento emocional fue del 28,1 % (intervalo de confianza del 95 %, IC: 21,5-33,5), un nivel alto de despersonalización fue del 16,4 % (IC del 95 %: 10,1-22,9) y un nivel alto de disminución de la realización personal fue del 31,9 % (IC del 95 %: 21,7-39,1).

**Conclusión:**

La considerable prevalencia del desgaste profesional entre los profesionales de la atención primaria en los países de ingresos bajos y medios tiene implicaciones para la seguridad de los pacientes, la calidad de la atención y la planificación del personal. Se necesitan más estudios transversales para ayudar a identificar soluciones fundamentadas en la evidencia, en especial en África y Asia Sudoriental.

## ملخص

**الغرض:**

تقدير مدى انتشار الإرهاق بين أخصائيي الرعاية الصحية الأولية في الدول ذات الدخل المنخفض والدخل المتوسط، وتحديد العوامل المرتبطة بالإرهاق.

الطريقة قمنا بالبحث بشكل منهجي في تسع قواعد للبيانات حتى فبراير/شباط 2022، لتحديد الدراسات التي تقوم بالاستقصاء في الإنهاك لدى أخصائيي الرعاية الأولية في الدول ذات الدخل المنخفضة والدخل المتوسطة. لم تكن هناك قيود تتعلق باللغة، وقمنا بتضمين دراسات قائمة على الملاحظات. قام اثنان من المراجعين المستقلين بإكمال عمليات الفحص، وتحديد الدراسة، واستخراج البيانات، وتقييم الجودة. تم استخدام التحليل التلوي للتأثيرات العشوائية لتقدير مدى انتشار الإرهاق الكلي كما تم تقييمه باستخدام المقاييس الفرعية Maslach Burnout Inventory للإرهاق العاطفي، وفقدان الشخصية، والإنجاز الشخصي. نحن نقوم بالإبلاغ عن العوامل المرتبطة بالإرهاق عن طريق السرد.

النتائج أسفر البحث عن 1568 مقالةً. بعد الاختيار، تم تضمين 60 دراسةً من 20 دولة في المراجعة السردية، وتضمين 31 دراسة في التحليل التلوي. جمعت ثلاث دراسات البيانات أثناء جائحة مرض فيروس كورونا 2019، ولكنها قدمت أدلة محدودة على تأثير المرض على الإرهاق. تراوح معدل الانتشار الكلي للإرهاق في نقطة واحدة من 2.5 إلى 87.9% (43 دراسة). في التحليل التلوي (31 دراسة)، كان معدل الانتشار المجمع لمستوى عالٍ من الإرهاق العاطفي هو 28.1% (بفاصل ثقة مقداره 95%: 21.5 إلى 33.5)، ومستوى عالٍ من فقدان الشخصية هو 16.4% (بفاصل ثقة مقداره 95%: 10.1 إلى 22.9)، ومستوى عالٍ من الإنجاز الشخصي هو 31.9% (بفاصل ثقة مقداره 95%: 21.7 إلى 39.1).

الاستنتاج إن الانتشار الكبير للإرهاق بين أخصائيي الرعاية الصحية الأولية في الدول ذات الدخل المنخفض والدخل المتوسط، له آثار على سلامة المرضى، وجودة الرعاية، وتخطيط القوى العاملة. هناك حاجة إلى مزيد من الدراسات المقطعية للمساعدة في تحديد الحلول القائمة على الأدلة، وخاصة في أفريقيا وجنوب شرق آسيا.

## 摘要

**目的:**

评估低收入和中等收入国家初级医疗护理工作者的职业倦怠率，并确定引起职业倦怠的相关因素。

**方法:**

我们系统地搜索了截至 2022 年 2 月的九个数据库，以确定调查低收入和中等收入国家初级医疗护理工作者职业倦怠情况的研究。搜集的研究没有语言限制，而且我们纳入了观察性研究。由两位独立评审员完成了筛查、研究选择、数据提取和质量评估。我们采用随机效应元分析法来评估整体职业倦怠率，并使用《马氏职业倦怠量表》作为子量表来评估情绪疲劳、人格解体和个人成就感的情况。我们对于职业倦怠相关的因素进行了叙述性报告。

**结果:**

搜索出 1568 篇论文。经过筛选，来自 20 个国家的 60 项研究被纳入叙述性回顾，31 项研究被纳入元分析。有三项研究收集了新型冠状病毒肺炎大流行期间的数据，但该疾病对职业倦怠的影响方面的证据有限。职业倦怠的总体单点流行率范围为 2.5% 至 87.9%（43 项研究）。在元分析（31 项研究）中，严重级别情绪疲劳的综合流行率为 28.1% （95% 置信区间，CI：21.5–33.5），严重级别人格解体的流行率为 16.4% (95% CI: 10.1-22.9），个人成就感严重缺失的流行率为 31.9% (95% CI: 21.7-39.1）。

**结论:**

低收入和中等收入国家初级医疗护理工作者的高职业倦怠率对患者安全、护理质量和人力规划会产生影响。需要开展进一步的横断面研究来帮助确定有证据支持的解决方案，特别是在非洲和东南亚地区。

## Резюме

**Цель:**

Оценить распространенность профессионального выгорания среди специалистов первичной медико-санитарной помощи в странах с низким и средним уровнем дохода, а также выявить факторы, связанные с профессиональным выгоранием.

**Методы:**

Авторы провели систематический поиск по девяти базам данных, собранных до февраля 2022 года, и выявили исследования о профессиональном выгорании среди специалистов первичной медико-санитарной помощи в странах с низким и средним уровнем дохода. Языковых ограничений не было. Были включены обсервационные исследования. Два независимых эксперта провели скрининговое обследование, отбор исследований, извлечение данных и оценку качества. Для оценки общей распространенности профессионального выгорания использовался метаанализ случайных эффектов с помощью подшкал опросника выгорания Маслач для определения эмоционального истощения, деперсонализации и личных достижений. Авторы описательно сообщают о факторах, связанных с профессиональным выгоранием.

**Результаты:**

В результате поиска обнаружено 1568 статей. После завершения отбора 60 исследований из 20 стран были включены в описательный анализ, а 31 – в метаанализ. Три исследования собрали данные во время пандемии коронавирусной инфекции 2019 года, но представили ограниченные данные о влиянии болезни на профессиональное выгорание. Общая распространенность профессионального выгорания колебалась от 2,5 до 87,9% (43 исследования). В метаанализе (31 исследование) общая распространенность высокого уровня эмоционального истощения составила 28,1% (95%-й ДИ: 21,5–33,5), высокий уровень деперсонализации составил 16,4% (95%-й ДИ: 10,1–22,9), а высокий уровень снижения личных достижений составил 31,9% (95%-й ДИ: 21,7–39,1).

**Вывод:**

Значительная распространенность эмоционального выгорания среди специалистов первичной медико-санитарной помощи в странах с низким и средним уровнем дохода влияет на безопасность пациентов, качество медицинской помощи и планирование трудовых ресурсов. Необходимы дальнейшие поперечные исследования для поиска основанных на фактических данных решений, особенно в Африке и Юго-Восточной Азии.

## Introduction

Burnout is defined as a form of chronic occupational stress consisting of three dimensions: (i) exhaustion; (ii) depersonalization or cynicism; and (iii) feelings of inefficacy.^1^ Although the burden of burnout in high-income countries is well established, less is known about low- and middle-income countries. Knowledge about burnout is important because of its substantial consequences.^2^^–^^6^ Among health-care professionals, burnout has been associated with patient safety concerns and poor quality of care.^2^ There is also an impact on physical and mental health and an increase in sick leave, staff turnover and emigration rates.^3^^–^^7^ Moreover, burnout can increase direct and indirect costs.^6^^,^^8^

Studies have demonstrated that the prevalence of burnout differs between countries and that it may be difficult to generalize research findings from high-income countries to low- and middle-income countries because of cultural differences that may affect factors associated with burnout and its prevalence.^9^^,^^10^ Additionally, the imbalance between job demands and the resources available underlies the etiology of burnout;^11^ this imbalance may differ substantially between low- and middle-income countries and high-income countries. Moreover, the coronavirus disease 2019 (COVID-19) pandemic changed the health-care landscape in many countries and introduced additional stressors, such as staff redeployment and the fear of infection.^12^ The impact of the pandemic on the prevalence of burnout and the possibility that factors associated with the pandemic may differ across regions warrants investigation.

In 2019, the World Health Organization (WHO) identified good primary health care as fundamental for achieving universal health coverage (UHC), a WHO strategic priority.^13^ UHC refers to the provision of universal, cost-effective health services that can be accessed without financial hardship.^13^ However, as observed, “health services are only as effective as the persons responsible for delivering them.”^14^ Thus, the physical and mental well-being of primary health-care professionals is crucial for achieving UHC. There is clear evidence from high-income countries that the prevalence of burnout in health-care professionals differs according to specialty and that the risk may be higher in primary care.^15^ Having a good estimate of the prevalence of burnout in primary health-care professionals in low- and middle-income countries is important because this information will provide the first step in identifying ways to mitigate the impact of burnout and to develop culturally and organizationally appropriate interventions.

The aims of this review, therefore, were: (i) to provide a comprehensive overview and meta-analysis of the prevalence of burnout among primary health-care professionals in low- and middle-income countries; (ii) to explore factors associated with burnout in these countries; and (iii) to compare data on burnout collected during the COVID-19 pandemic and the pre-pandemic period.

## Methods

When performing this review, we followed the preferred reporting items for systematic reviews and meta-analyses.^16^ We conducted an initial systematic search in nine electronic databases from database inception to 16 November 2020: (i) MEDLINE®; (ii) CINAHL; (iii) PsycInfo; (iv) APA PsycArticles®; (v) AMED; (vi) Embase®; (vii) Web of Science Core Collection; (viii) Global Index Medicus; and (ix) CNKI. Searches were updated on 11 February 2022. Reference lists were hand searched. Box 1 (available at: https://www.who.int/publications/journals/bulletin/) presents the combination of search terms. The full search strategy conducted on MEDLINE® via EBSCOhost (EBSCO Information Services, Ipswich, United States of America, is extensive; details are available from the data repository.^17^ There were no search limitations.

Box 1 **Search term combinations used in the meta-analysis of burnout in primary health-care professionals in low- and middle-income countries up to 2022**

- primary health-care providers, such as (“general practi*”), (“family physician”), (“primary N2 care”), (“community health care”), (“community health work*”) and (“community N3 nurse”);
- with terms for burnout, such as (“burnout”), (“compassion fatigue”), (“emotional exhaustion”), (disengage*), (“occupation* N3 stress*”) and (“work* N3 stress*); and
- terms for low- and middle-income countries that included each country name along with additional terms such as (MH “Developing Countries”), (“middle income*” W0 (countr* OR nation OR nations OR econom*)), (“low* income” W0 (countr* OR nation OR nations OR econom*)), (“third world” W0 (countr* OR nation OR nations OR econom*)), (“less* developed” W0 (countr* OR nation OR nations OR econom*)), (Africa*), (West* W0 Asia*), ((South OR Southern) W0 Asia*), ((Latin OR Central OR South) W0 America*), ((Middle OR Far) W0 East) and (Caribbean* OR “West Indies*).

Study eligibility criteria are listed in Box 2 (available at: https://www.who.int/publications/journals/bulletin/). We included studies in the meta-analysis if the Maslach Burnout Inventory was used as the measurement tool and prevalence estimates were reported for each of the following three subscales:^19^ (i) emotional exhaustion; (ii) depersonalization; and (iii) personal accomplishment. Low- and middle-income countries were defined by the World Bank’s 2020 income classification.^18^ We exported search results to Rayyan Intelligent Systematic Review (Rayyan Systems Inc., Cambridge, USA) for de-duplication and screening. One reviewer completed title screening and a second reviewer independently screened 10% of titles for comparability. Two reviewers independently completed abstract and full text screening; disagreements were resolved through discussion. We developed the protocol for this systematic review and meta-analysis a priori and registered with PROSPERO (CRD42020221336).^20^

Box 2 **Study eligibility criteria, meta-analysis of burnout in primary health-care professionals in low- and middle-income countries up to 2022**

**Inclusion criteria**

- Study design: cross-sectional or cohort study
- Study setting: low- or middle-income country, as defined by the World Bank’s 2020 income classification^18^
- Study population: primary health-care professionals working in community settings
- Primary outcome of study: burnout prevalence as assessed using a validated burnout measurement tool or by self-report
- Secondary outcome of study: factors associated with burnout

**Exclusion criteria**

- Duplicates of publications or secondary research, such as narrative reviews or opinion pieces
- Studies in high-income countries, as defined by the World Bank’s 2020 income classification^18^
- Studies involving or including hospital-based secondary care professionals or specialists that report no separate data for primary care practitioners
- Studies on medical students
- Research on anxiety, depression or occupational stress that does not have a specific focus on burnout

Data extracted included: (i) study author; (ii) year of publication; (iii) country; (iv) region; (v) country income classification; (vi) study design; (vii) study participants; (viii) sampling method; (ix) sample size; (x) participants’ mean age; (xi) percentage of female participants; (xii) measurement tool; (xiii) prevalence of overall burnout; and (xiv) prevalence of burnout according to measurement tool subscales and to any associated factors. Two reviewers extracted data independently using a form developed and piloted for the study and at the same time performed a quality assessment using Hoy et al.’s risk-of-bias tool for prevalence studies,^21^ details available from the data repository.^17^ Disagreements were resolved through discussion. We translated non-English studies using Google Translate (Google LLC, Mountain View, USA).

### Data analysis

Study characteristics, the burnout prevalence range and factors associated with burnout are reported narratively for all eligible studies. A random-effects model was used for the meta-analysis. We performed the analysis with MetaXL v. 5.3 (EpiGear International Pty Ltd) using the double arcsine transformation variant for the meta-analysis of prevalence.^22^ We calculated pooled prevalence estimates for each score category (i.e. high, moderate and low) in the three Maslach Burnout Inventory subscales and reported with 95% confidence intervals (CIs). Standard values for the subscale score categories are listed in Table 1. Subgroup analyses were carried out for different professional groups. Study heterogeneity was assessed by inspecting forest plots and by calculating *I^2^* – an *I^2^* greater than 60% indicated a high degree of heterogeneity. Publication bias was assessed using Doi plots and the LFK index.^23^

**Table 1:** Definitions of low, moderate and high Maslach Burnout Inventory subscale scores, meta-analysis of burnout in primary health-care professionals in low- and middle-income countries up to 2022

Maslach Burnout Inventory subscale	Subscale score, score category		
	Low	Moderate	High
Emotional exhaustion	≤ 16	17–26	≥ 27
Depersonalization	≤ 5	6–9	≥ 10
Personal accomplishment	≤ 33	34–39	≥ 40

## Results

**Fig. 1:**
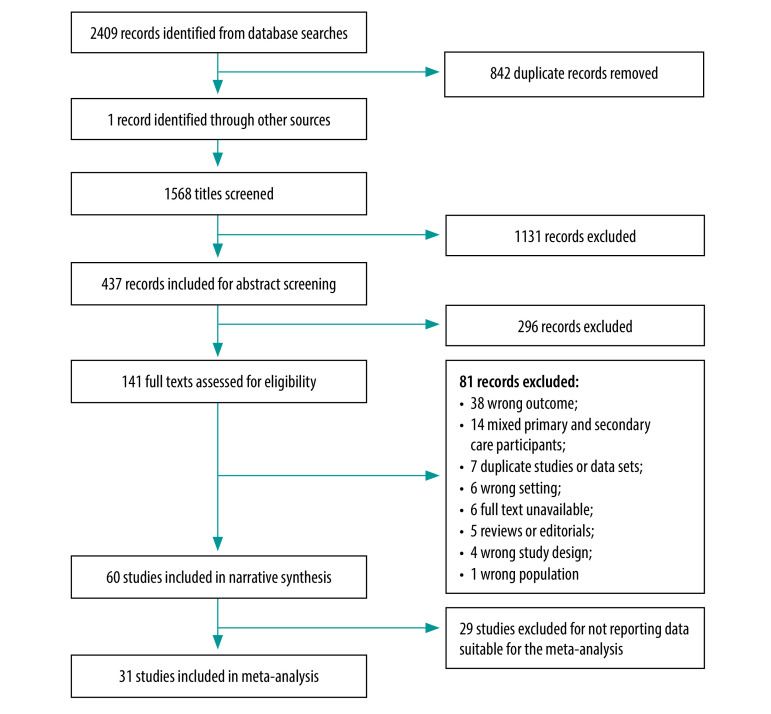
Selection of studies, meta-analysis of burnout in primary health-care professionals in low- and middle-income countries up to 2022

The literature searches generated a total of 1568 unique articles once duplicates were removed (Fig. 1). After screening, we included 60 studies in the narrative review and 31 studies in the meta-analysis.

### Study characteristics

The 60 studies in the narrative review included a total of 61 089 primary health-care professionals from 20 low- and middle-income countries. The sample size ranged from 28 to 21 759. There were 61 different country data sets as one study included two countries: Bulgaria and Turkey.^24^ The greatest number of studies came from Brazil (18 studies),^25^^–^^42^ followed by China (10 studies),^43^^–^^52^ and Mexico (6 studies).^53^^–^^58^ Every WHO region was represented, with the greatest number of studies (25 studies) coming from the Region of the Americas. Box 3 (available at: https://www.who.int/publications/journals/bulletin/) summarizes the geographical spread of studies.

Box 3 **Data sets, by WHO region and country, meta-analysis of burnout in primary health-care professionals in low- and middle-income countries up to 2022**

**Region of the Americas (25 studies)**

18 studies from Brazil; six studies from Mexico; and one study from Cuba.

**European Region (11 studies)^a^**

Five studies from Turkey; two studies from Bosnia and Herzegovina; two studies from Serbia; one study from Bulgaria; and one study from the Russian Federation.

**Western Pacific Region (10 studies)**

10 studies from China.

**Eastern Mediterranean Region (seven studies)**

Four studies from the Islamic Republic of Iran; one study from Egypt; one study from Iraq; and one study from West Bank and Gaza Strip.

**African Region (six studies)**

Two studies from South Africa; one study from Cameroon; one study from Ethiopia; one study from Uganda; and one study from Zambia.

**South-East Asia Region (two studies)**

One study from India; and one study from Thailand.

WHO: World Health Organization.^a^ One study included data from Bulgaria and Turkey.

According to the World Bank’s 2020 income classification, most (54) data sets in this review were from upper-middle-income countries. Five were from lower-middle-income countries;^59^^–^^63^ two were from low-income countries.^64^^,^^65^ There were 20 non-English language studies: 11 Portuguese;^26^^–^^30^^,^^32^^,^^33^^,^^36^^,^^37^^,^^39^^,^^40^ eight Spanish;^35^^,^^53^^–^^58^^,^^66^ and one French.^59^ All Chinese publications that fulfilled our inclusion criteria were available in English. Overall, 54 studies reported participants according to gender and 31 reported their mean age, which ranged from 28 (standard deviation, SD: 2.59) to 47 (SD: 8.48) years. Table 2 (available at: https://www.who.int/publications/journals/bulletin/) lists the different types of health-care worker included in the studies.

**Table 2:** Study participant type, meta-analysis of burnout in primary health-care professionals in low- and middle-income countries up to 2022

Study participants	No. (%) of studies (*n* = 60)
Family physicians	20 (33.3)
Mixed primary health-care professionals	18 (30.0)
Community nurses and nursing assistants	12 (20.0)
CHWs	6 (10.0)
Community pharmacists	2 (3.3)
Community midwives	1 (1.7)
Community oral health team members	1 (1.7)

CHW: community health worker.

The measurement tool used by 47 of the 60 studies was the Maslach Burnout Inventory. In the remaining 13 studies, the tool used was either: the Spanish Burnout Inventory;^29^^,^^36^^,^^39^ the Compassion Fatigue Questionnaire;^67^ the Professional Quality of Life scale;^65^ the Oldenburg Burnout Inventory;^40^ a short, validated questionnaire based on the Maslach Burnout Inventory;^66^ the Burnout Measure;^68^^,^^69^ the Copenhagen Burnout Inventory;^63^ the Burnout Characterization Scale;^42^ Emotional Burnout Diagnostics by Boyko V.V.;^70^ or a single-item scale.^61^ Only three studies reported collecting data during the COVID-19 pandemic:^68^^–^^70^ two were conducted in Turkey and used the Burnout Measure (short version);^68^^,^^69^ and one was conducted in the Russian Federation and used Emotional Burnout Diagnostics.^70^ One study included family medicine residents,^68^ one nurses,^70^ and one community pharmacists.^69^ Table 3 summarizes the studies’ characteristics.

**Table 3:** Study characteristics, meta-analysis of burnout in primary health-care professionals in low- and middle-income countries up to 2022

Study and year	Country	Type of participant	Burnout measurement tool	No. of participants	Mean age of participants, years	Proportion of female participants, %	Overall burnout prevalence, %	Prevalence of burnout by MBI subscale score category, (%)^a^		
								Emotional exhaustion	Depersonalization	Personal accomplishment
Putnik 2011^71^	Serbia	Family physicians	MBI–General Survey	373	47	84	ND	High (48.3); moderate (34.0)	High (12.9); moderate (32.7)	High (5.1); moderate (16.9)
Mandengue 2017^59^	Cameroon	Family physicians	MBI–Human Services Survey	85	ND	48.2	42.4	High (11.8); moderate (18.8)	High (10.6); moderate (31.8)	High (30.6); moderate (29.4)
López-León 2007^56^	Mexico	Family physicians	MBI	131	46.4 (SD: 6.3)	42	39.7	High (26.0); moderate (22.1)	High (19.8); moderate (12.3)	High (8.4); moderate (14.5)
Lesić 2009^72^	Serbia	Family physicians	MBI	38	42.2 (SD: 10.7)	79.0	ND	High (29.0); moderate (45.2)	High (11.1); moderate (27.8)	High (24.2); moderate (27.3)
Kotb 2014^60^	Egypt	Family physicians	MBI	31	ND	80	41.94	ND	ND	ND
Kosan 2019^73^	Turkey	Family physicians	MBI	385 (139 in 2008 and 246 in 2012)	2008: 30 (SD: 5.13); 2012: 34.05 (SD: 5.78)	64.2 (48.9 in 2008 and 72.8 in 2012)	ND	2008: high (0.7) and moderate (24.5); 2012: high (9.3) and moderate (21.5)	2008: high (4.3) and moderate (18.0); 2012: high (4.5) and moderate (19.9)	2008: high (76.3) and moderate (21.6); 2012: high (79.3) and moderate (17.4)
Gan 2019^45^	China	Family physicians	MBI–Human Services Survey	1015	ND	ND	35.0 (high on one MBI subscale); 21.0 (high on two subscales); 2.5 (high on three subscales)	High (24.83); moderate (23.25)	High (6.21); moderate (12.0)	High (33.99); moderate (20.0)
Charoentanyarak 2020^74^	Thailand	Family physician residents	MBI–Human Services Survey	149	28.29 (SD: 2.59)	67.1	10.7	High (33.56); moderate (30.87)	High (14.09); moderate (27.52)	High (1.34); moderate (2.68)
Cetina-Tabares 2006^55^	Mexico	Family physicians	MBI	93	44	46.2	20.5 (high on three subscales); 29.0 (moderate on three MBI subscales)	ND	ND	ND
Stanetić 2013^75^	Bosnia and Herzegovina	Family physicians	MBI–Human Services Survey	239	ND	83.3	ND	High (46.0); moderate (28.9)	High (21.3); moderate (31.8)	High (22.2); moderate (34.7)
Soler 2008^24^	Bulgaria and Turkey	Family physicians	MBI–Human Services Survey	69 in Bulgaria and 112 in Turkey	ND	ND	ND	Bulgaria: high (62.3); Turkey: high (15.2)	Bulgaria: high (30.4); Turkey: high (15.2)	Bulgaria: high (18.8); Turkey: high (69.4)
Aranda 2004^53^	Mexico	Family physicians	MBI–Human Services Survey	163	47	36.2	42.3	High (16.0); moderate (16.0)	High (1.8); moderate (5.5)	High (6.7); moderate (8.6)
Aranda-Beltrán 2005^54^	Mexico	Family physicians	MBI	197	ND	37.1	41.8	High (13.3); moderate (17.9)	High (2.0); moderate (6.6)	High (6.6); moderate (7.7)
Al Dabbagh 2019^76^	Iraq	Family physicians	MBI	134	ND	64.8	30.6 (high); 50.0 (moderate)	High (68.7); moderate (11.9)	High (26.1); moderate (28.4)	High (41.1); moderate (26.1)
Ahmadpanah 2015^77^	Iran (Islamic Republic of)	Family physicians	MBI	100	32.90 (SD: 5.06)	29	ND	High (15.4)	High (14.5)	High (10.2)
Aguilera 2010^57^	Mexico	Family physicians	MBI–Human Services Survey	233	44.4 (SD: 7.18)	40.3	41.6	High (31.7)	High (15.0)	High (15.9)
Račić 2019^67^	Bosnia and Herzegovina	Family physicians	Compassion fatigue questionnaire	120	ND	80	75 (moderate)	ND	ND	ND
Rossouw 2013^78^	South Africa	Family physicians	MBI	132	ND	ND	ND	High (53)	High (64)	High (43)
Çevik 2021^68^^b^	Turkey	Family medicine residents	Burnout Measure (short version)	477	Median: 28 (range: 24–54)	61.2	25.8 (moderate); 24.1 (severe); 23.3 (very severe)	ND	ND	ND
Zhang 2021^52^	China	Family physicians	MBI–General Survey (Chinese version)	2 693	44.64 (SD: 7.25)	35.6	65.2	High (30.1); moderate (24.2)	High (22.2); moderate (11.7)	High (48.3); moderate (13.3)
Engelbrecht 2008^79^	South Africa	Community nurses	MBI	542	ND	ND	ND	High (68.7); moderate (30.9)	High (85.1); moderate (12.9)	High (8.3); moderate (91.0)
Hu 2015^44^	China	Community nurses	MBI	420	ND	100	86.2	ND	ND	ND
Alshawish 2020^62^	West Bank and Gaza Strip	Community nurses and midwives	MBI	207	ND	91.3	10.6	High (36.7); moderate (17.9)	High (14.0); moderate (20.8)	High (17.9); moderate (19.3)
Merces 2017^32^	Brazil	Community nurses	MBI–Human Services Survey	60	39.55 (SD: 10.38)	95	58.3 (high on at least one MBI subscale); 16.7 (high on all three subscales)	High (18.3); moderate (43.3)	High (48.3); moderate (41.7)	High (56.6); moderate (41.7)
Merces 2016^30^	Brazil	Community nurses	MBI	28	39.1 (SD: 9.6)	100	7.1	High (28.6); moderate (39.3)	High (21.5); moderate (32.1)	High (46.4); moderate (50.0)
Barbosa Ramos 2019^37^	Brazil	Community nurses	MBI	52	ND	100	ND	High (15.4); moderate (34.6)	High (13.5); moderate (34.6)	High (23.1); moderate (21.2)
Merces 2016^31^	Brazil	Community nurses	MBI	189	ND	96.8	10.6	High (20.6); moderate (40.7)	High (31.7); moderate (39.2)	High (48.1); moderate (49.2)
Lorenz 2018^34^	Brazil	Community nurses	MBI	168	ND	88.4	ND	High (28.0); moderate (37.5)	High (32.1); moderate (33.9)	High (38.7); moderate (33.3)
Holmes 2014^26^	Brazil	Community nurses	MBI	45	ND	100	11.1	High (53.3); moderate (20.0)	High (11.1); moderate (28.9)	High (11.1); moderate (48.9)
Merces 2020^38^	Brazil	Community nurses	MBI–Human Services Survey	1125	37.1 (SD: 9.6)	87.9	18.3	High (28.1); moderate (41.1)	High (44.5); moderate (35.9)	High (60.2); moderate (36.2)
Garcia 2021^42^	Brazil	Community nurses	Burnout characterization scale	122	45.2 (SD: 9.8)	94.3	ND	High (27.9); moderate (37.7)	High (25.4); moderate (41.8)^c^	High (25.4); moderate (47.5)^d^
Seluch 2021^70^^b^	Russian Federation	Community nurses	Emotional Burnout Diagnostics by Boyko V.V.	60	40.86	100	50	ND	ND	ND
Silveira 2014^29^	Brazil	Mixed primary health-care professionals	CESQT	217	ND	88.9	18 (profile 1); 11 (profile 2)	ND	ND	ND
da Silva 2008^25^	Brazil	Mixed primary health-care professionals	MBI	141	38.9 (SD: 11.4)	92.2	24.1	Moderate or high (70.9)	Moderate or high (34.0)	Moderate or high (47.5)
Selamu 2019^64^	Ethiopia	Mixed primary health-care professionals	MBI–Human Services Survey	136	ND	61	3.8 (at baseline); 4.6 (at 6-month follow-up)	High (7.7 at baseline; 7.5 at 6-month follow-up)	ND	High (43.7 at baseline; 48.5 at 6-month follow-up)
Hernández 2003^66^	Cuba	Mixed primary health-care professionals	Short questionnaire of burnout	144	ND	77.1	43.8 (doctors); 27.3 (nurses)	ND	ND	ND
Ran 2020^47^	China	Mixed primary health-care professionals	MBI–General Survey	1 279	ND	66.5	18.69	ND	ND	ND
Pinheiro 2020^39^	Brazil	Mixed primary health-care professionals	CESQT	344	40 (SD: 9.7)	88.7	14.4 (profile 1); 44.5 (profile 2)	ND	ND	ND
Mao 2020^48^	China	Mixed primary health-care professionals	MBI	663	ND	44.5	ND	High (24.1); moderate (14.6)	High (15.7); moderate (7.4)	High (34.7); moderate (15.8)
Lima 2018^33^	Brazil	Mixed primary health-care professionals	MBI–Human Services Survey	153	45 (SD: 9.78)	82.4	51	ND	ND	ND
Li 2019^46^	China	Mixed primary health-care professionals	MBI–Human Services Survey	951	ND	65.1	ND	High (33.1); moderate (32.9)	High (8.8); moderate (19.8)	High (41.43); moderate (20.5)
Kruse 2009^61^	Zambia	Mixed primary health-care professionals	Single-item scale	483	37 (IQR: 31–45)	87	51.2	ND	ND	ND
Hernández-Vargas 2009^58^	Mexico	Mixed primary health-care professionals	MBI	276	ND	ND	ND	High (34.8); moderate (30.1)	High (35.1); moderate (19.6)	High (36.2) Moderate (30.4)
Xu 2020^49^	China	Mixed primary health-care professionals	MBI	15 627	ND	66.2	3.3 (high); 47.6 (moderate)	ND	ND	ND
Wang 2020^43^	China	Mixed primary health-care professionals	MBI	1 148	ND	64.72	ND	High (27.66)	High (6.06)	High (38.74)
Tomaz 2020^40^	Brazil	Mixed primary health-care professionals	Oldenburg Burnout Inventory	94	40.9 (SD: 9.6)	84	38.3	High (21.3)	ND	ND
de Souza Filho 2019^36^	Brazil	Mixed primary health-care professionals	CESQT	248	40.75 (SD: 9.66)	91.1	24.2 (profile 1); 8.5 (profile 2)	ND	ND	ND
da Silva 2021^41^	Brazil	Mixed primary health-care professionals	MBI	2 940	36.7 (SD: 9.6)	90.5	11.4 (severe)	High (39.7); moderate (24.9)	High (11.8); moderate (24.5)	High (18.3); moderate (27.2)
Lu 2020^50^	China	Mixed primary health-care professionals	MBI	21 759	35	70.0	50.1 (total); 3.0 (severe); 47.1 (moderate)	ND	ND	ND
Yan 2021^51^	China	Mixed primary health-care professionals	MBI (Chinese version)	1 214	40.26 (SD: 8.61)	55	11.3 (severe); 37.6 (moderate)	ND	ND	ND
Malakouti 2011^80^	Iran, (Islamic Republic of)	CHWs	MBI	212	35.1 (SD: 7.2)	70.1	1.1 (high); 16.6 (moderate)	High (12.3); moderate (15.1)	High (5.3); moderate (8.0)	High (43.7); moderate (19.0)
Mota 2014^28^	Brazil	CHWs	MBI	222	ND	87.8	29.3	Moderate or high (57.7)	Moderate or high (51.8)	Moderate or high (59.0)
Martins 2014^27^	Brazil	CHWs	MBI	107	ND	ND	41.6	High (20.6); moderate (52.3)	High (21.1); moderate (50.0)	High (20.6); moderate (55.4)
Bijari 2016^81^	Iran (Islamic Republic of)	CHWs	MBI	423	39 (SD: 8.4)	57.9	5.7 (high on all three MBI subscales); 28.8 (high on either emotional exhaustion or depersonalization subscale)	High (17.7); moderate (13.7)	High (6.4); moderate (10.4)	High (53.0); moderate (18.2)
Amiri 2016^82^	Iran (Islamic Republic of)	CHWs	MBI	548	35.8 (SD: 7.5)	71	5.5 (high); 52.7 (moderate)	High (17.3); moderate (18.4)	High (8.8); moderate (10.0)	High (33.9); moderate (15.7)
Pulagam 2021^63^	India	CHWs	Copenhagen Burnout Inventory	150	ND	100	Personal burnout: 8.0 (high) and 30 (moderate); work burnout: 8.7 (high) and 24.7 (moderate); client burnout: 6.7 (high) and 23.3 (moderate)	ND	ND	ND
Muliira 2016^65^	Uganda	Midwives	Professional Quality of Life scale	224	34 (SD: 6.3)	79.5	10.3 (high); 87.9 (moderate)	ND	ND	ND
Maciel 2018^35^	Brazil	Community oral health team members	MBI–Human Services Survey	50	ND	72	ND	High (26); moderate (32)	High (16); moderate (26)	High (10); moderate (26)
Calgan 2011^83^	Turkey	Community pharmacists	MBI	251	42.06 (SD: 11.19)	58.6	ND	High (1.2); moderate (27.1)	High (0.8); moderate (13.9)	High (71.3) Moderate (24.7)
Okuyan 2021^69^^b^	Turkey	Community pharmacists	Burnout Measure (short version)	1 098	41	64.8	31.5	ND	ND	ND

CESQT: Spanish Burnout Inventory (*Cuestionario para la Evaluación del Síndrome de Quemarse por el Trabajo*); CHW: community health worker; IQR: interquartile range; MBI: Maslach Burnout Inventory; ND: not determined; SD: standard deviation.

^a^ Definitions of low, moderate and high score categories for the Maslach Burnout Inventory subscales of emotional exhaustion, depersonalization and personal accomplishment are listed in Table 1.

^b^ This study reported collecting data during the coronavirus 2019 pandemic.

^c^ Dehumanization was assessed instead of depersonalization.

^d^ Disappointment was assessed instead of personal accomplishment.

### Burnout prevalence

A single-point prevalence for overall burnout was reported by 43 studies, which used a range of different measurement tools and different definitions of burnout. Estimates ranged from 2.5% for severe burnout among family physicians in China to 87.9% for burnout among midwives in Uganda.^45^^,^^65^ In the three studies that collected data during the COVID-19 pandemic, the prevalence ranged from 31.5% in community pharmacists to 47.4% in family medicine residents (for severe or very severe burnout) to 50.0% in primary care nurses.^68^^–^^70^

Of 47 studies that reported burnout prevalence determined using the Maslach Burnout Inventory, 31 (involving 14 439 primary health-care professionals) contributed data suitable for the meta-analysis. The risk of bias was assessed as low for 18 of these studies and moderate for 13. No study had a high risk of bias. Of the two studies in the meta-analysis that were published during the pandemic, one collected data before the COVID-19 pandemic and one did not report dates for data collection. Table 4 shows the pooled prevalence of emotional exhaustion, depersonalization and reduced personal accomplishment across the 31 studies. The pooled prevalence was 28.1% for a high level of emotional exhaustion, 27.6% for a moderate level of emotional exhaustion, 16.4% for a high level of depersonalization, 22.7% for a moderate level of depersonalization, 31.9% for a high level of reduced personal accomplishment and 28.1% for a moderate level of reduced personal accomplishment. The combined estimated prevalence of a moderate or high level on each subscale was 55.7% for emotional exhaustion, 39.1% for depersonalization and 60.0% for reduced personal accomplishment. The *I*^2^ for these studies was 98% for the emotional exhaustion subscale and 99% for the depersonalization and personal accomplishment subscales, which indicate a high degree of heterogeneity. Forest plots for high scores on each subscale are available from the data repository.^17^

**Table 4:** Prevalence of burnout by Maslach Burnout Inventory subscale score category, meta-analysis of burnout in primary health-care professionals in low- and middle-income countries up to 2022

Maslach Burnout Inventory subscale score category^a^	Pooled prevalence, % (95% CI)^b^
**Emotional exhaustion**	
High	28.1 (21.5–33.5)
Moderate	27.6 (21.1–33.0)
Low	44.3 (36.6–49.9)
**Depersonalization**	
High	16.4 (10.1–22.9)
Moderate	22.7 (15.2–29.7)
Low	60.9 (50.5–67.6)
**Personal accomplishment**	
High	31.9 (21.7–39.1)
Moderate	28.1 (18.5–35.3)
Low	39.9 (28.7–47.0)

CI: confidence interval.

^a^ Definitions of low, moderate and high Maslach Burnout Inventory score categories are listed in Table 1.

^b^ Prevalence was pooled across 31 studies.

**Fig. 2:**
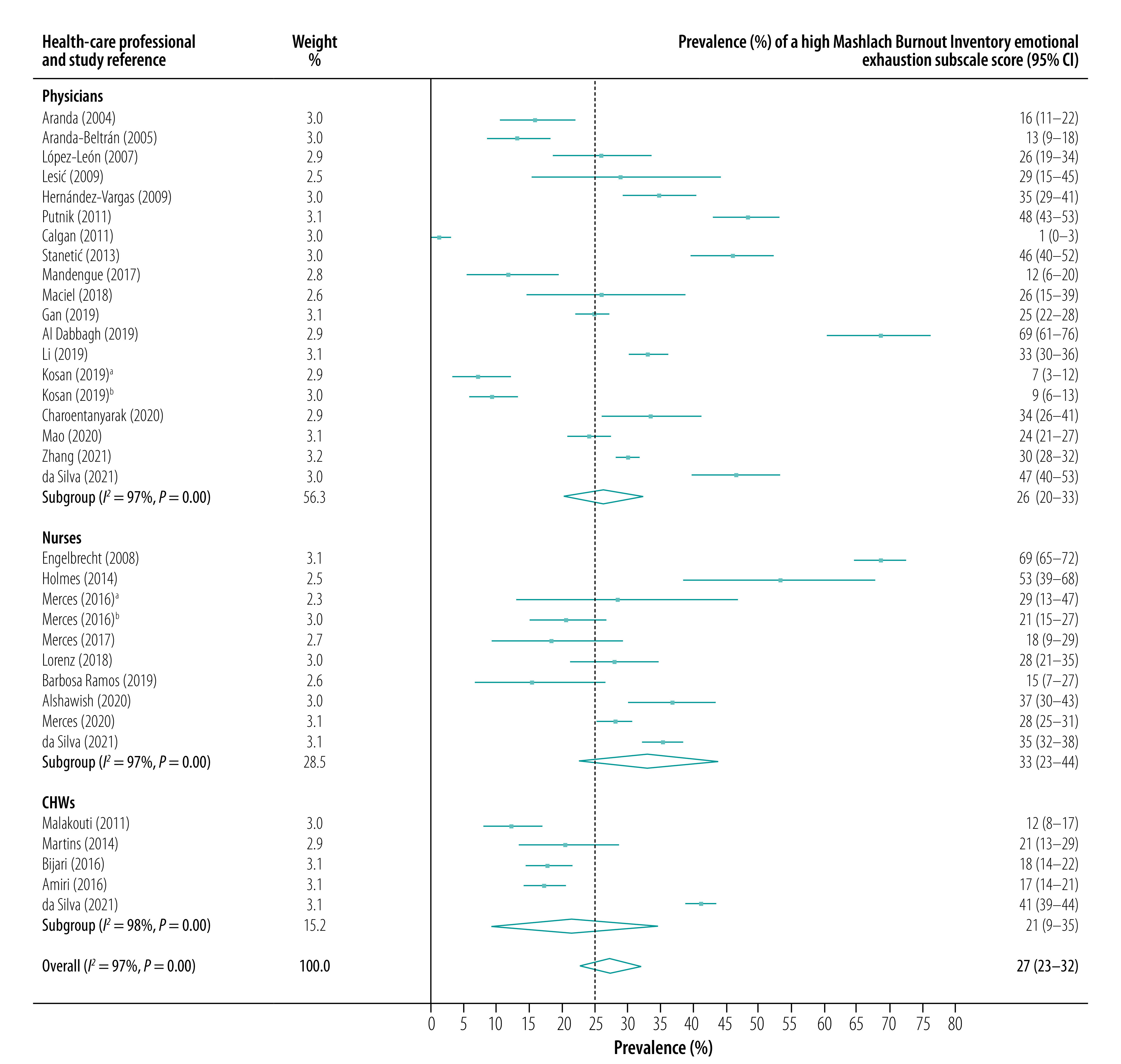
Prevalence of a high Maslach Burnout Inventory emotional exhaustion subscale score, by health-care professional type and study, meta-analysis of burnout in primary health-care professionals in low- and middle-income countries up to 2022

CHWs: community health workers; CI: confidence interval.

Notes: A high Maslach Burnout Inventory emotional exhaustion subscale score is defined in Table 1. For the Kosan 2019 study, data are represented separately for 2008 (Kosan 2019a) and 2012 (Kosan 2019b). The Merces 2016a study refers to the paper by Merces M, Carneiro e Cordeiro T, et al. 2016.^30^ The Merces 2016b study refers to the paper by Merces M, Silva D, et al. 2016.^31^

**Fig. 3:**
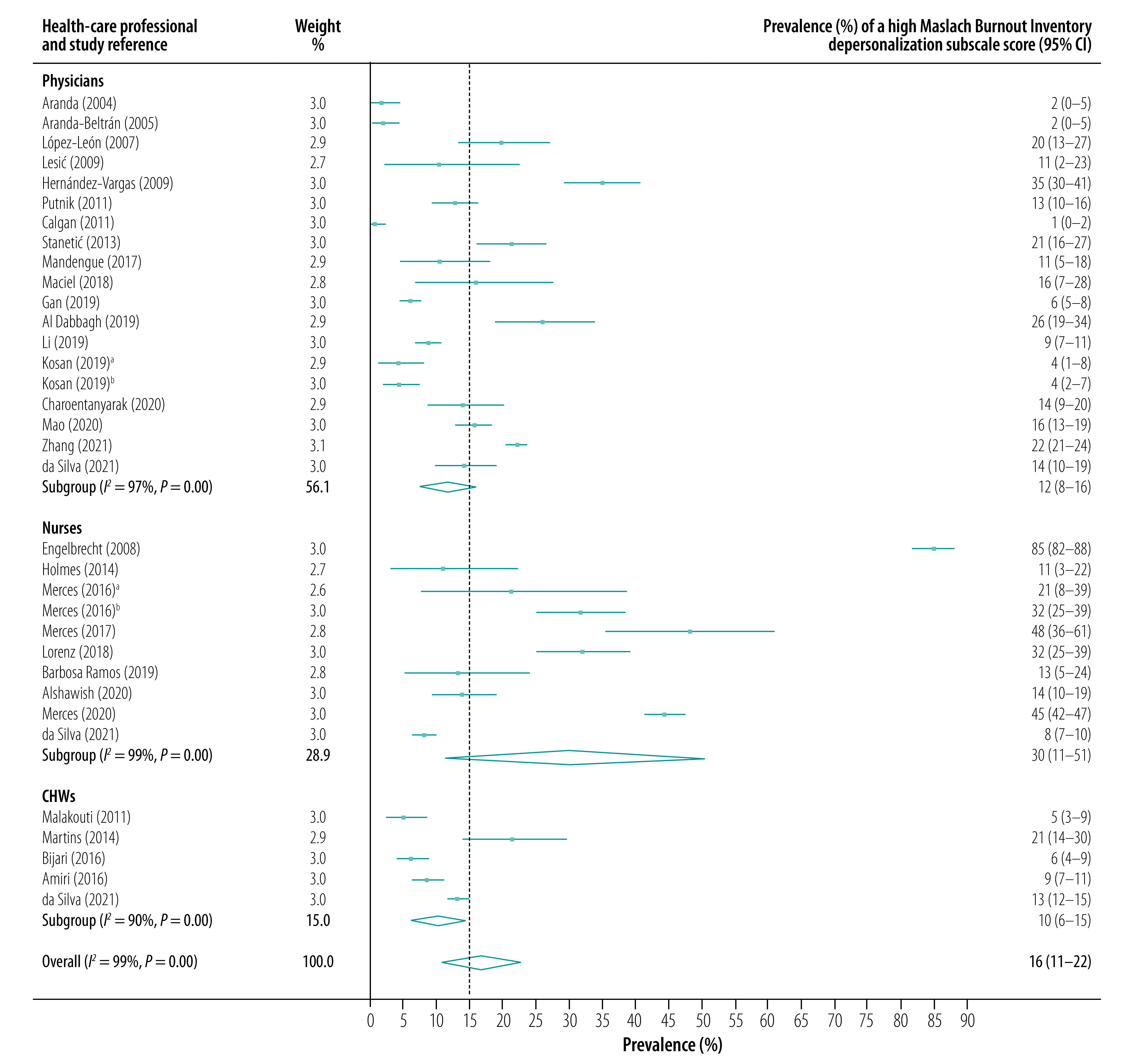
Prevalence of a high Maslach Burnout Inventory depersonalization subscale score, by health-care professional type and study, meta-analysis of burnout in primary health-care professionals in low- and middle-income countries up to 2022

CHWs: community health workers; CI: confidence interval.

Notes: A high Maslach Burnout Inventory depersonalization subscale score is defined in Table 1. For the Kosan 2019 study, data are represented separately for 2008 (Kosan 2019a) and 2012 (Kosan 2019b). The Merces 2016a study refers to the paper by Merces M, Carneiro e Cordeiro T, et al. 2016.^30^ The Merces 2016b study refers to the paper by Merces M, Silva D, et al. 2016.^31^

**Fig. 4:**
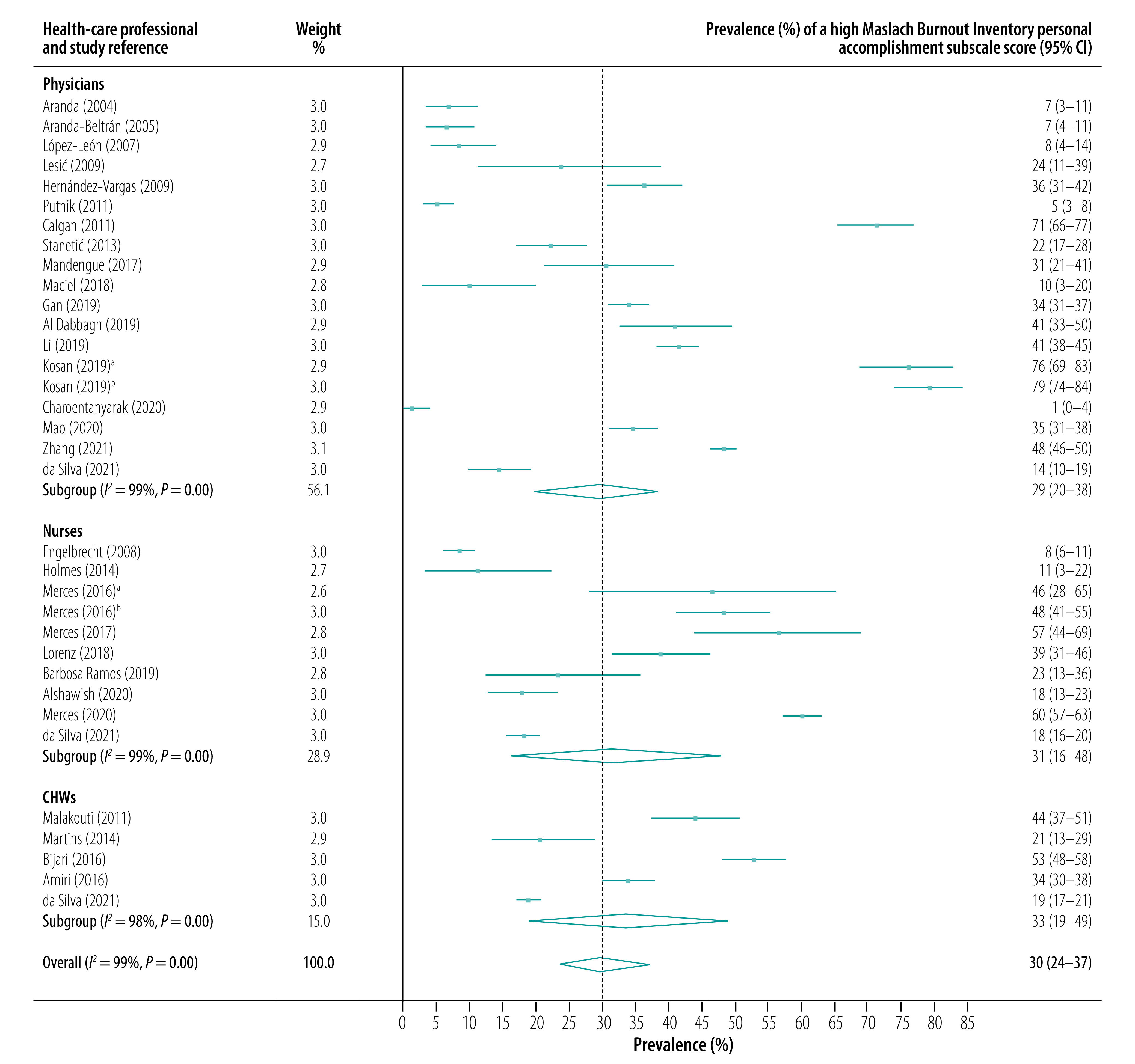
Prevalence of a high Maslach Burnout Inventory personal accomplishment subscale score, by health-care professional type and study, meta-analysis of burnout in primary health-care professionals in low- and middle-income countries up to 2022

CHWs: community health workers; CI: confidence interval.

Notes: A high Maslach Burnout Inventory personal accomplishment subscale score is defined in Table 1. A high score indicates reduced personal accomplishment. For the Kosan 2019 study, data are represented separately for 2008 (Kosan 2019a) and 2012 (Kosan 2019b). The Merces 2016a study refers to the paper by Merces M, Carneiro e Cordeiro T, et al. 2016.^30^ The Merces 2016b study refers to the paper by Merces M, Silva D, et al. 2016.^31^

The subgroup analysis showed that high scores for emotional exhaustion were most prevalent in community nurses (pooled prevalence: 33.1%; 95% CI: 22.7–44.0), followed by family physicians (pooled prevalence: 26.1%; 95% CI: 20.3–32.5) and community health workers (CHWs, pooled prevalence: 21.3%; 95% CI: 9.3–34.8). Depersonalization was also most prevalent among community nurses (pooled prevalence for a high score: 30.0%; 95% CI: 11.3–50.7), followed by family physicians (pooled prevalence: 11.5%; 95% CI: 7.8–16.0) and CHWs (pooled prevalence: 10.0%; 95% CI: 6.3–14.5). In contrast, reduced personal accomplishment was most prevalent in CHWs (pooled prevalence for a high score: 33.5%; 95% CI: 19.2–48.7), followed by nurses (pooled prevalence: 31.3%; 95% CI: 16.1–47.8) and family physicians (pooled prevalence: 28.7%; 95% CI: 19.7–38.4). Forest plots for the prevalence of high scores on the three Maslach Burnout Inventory subscales are presented in Fig. 2, Fig. 3 and Fig. 4.

### Factors associated with burnout

Demographic factors, such as sex, age, marital status and educational level, were associated with burnout in our review. Nine studies found a higher prevalence of burnout in women,^38^^,^^40^^,^^57^^,^^61^^,^^64^^,^^68^^,^^69^^,^^71^^,^^82^ six found a higher prevalence in men,^24^^,^^27^^,^^46^^,^^48^^–^^50^ and three found no significant sex difference.^45^^,^^56^^,^^60^ Burnout, specifically emotional exhaustion, was negatively associated with age in 10 studies,^24^^,^^27^^,^^41^^,^^49^^,^^50^^,^^60^^,^^61^^,^^69^^,^^73^^,^^76^ whereas four studies found a positive association.^64^^,^^75^^,^^81^^,^^82^ Burnout was positively associated with marriage in four studies,^40^^,^^44^^,^^60^^,^^65^ and with having children in four studies.^40^^,^^57^^,^^59^^,^^81^ In contrast, there was a positive association with unmarried status in eight studies,^45^^,^^49^^,^^50^^,^^53^^,^^54^^,^^68^^,^^76^^,^^83^ and with not having children in two.^29^^,^^68^ A high educational level was associated with burnout in eight studies.^44^^,^^46^^,^^48^^–^^50^^,^^53^^,^^54^^,^^81^

A heavy workload (including overtime, shift work and a high patient load) and having a second job were significantly associated with a high prevalence of burnout,^24^^,^^29^^,^^37^^,^^38^^,^^46^^,^^48^^–^^50^^,^^53^^,^^56^^,^^57^^,^^59^^–^^61^^,^^63^^,^^67^^,^^78^^,^^79^^,^^83^ as were exposure to violence and conflict at work.^38^^,^^44^^,^^45^^,^^56^^,^^59^^,^^74^^,^^79^ Other work-related factors included working in a rural or economically deprived setting,^27^^,^^38^^,^^41^^,^^64^^,^^67^ insufficient resources,^38^^,^^56^^,^^79^^,^^82^ COVID-19 exposure,^68^ inadequate personal protective equipment,^68^ a poor level of support,^46^^,^^48^^,^^58^ job insecurity,^58^^,^^63^^,^^64^ specific job tasks,^65^ and inadequate rest breaks or vacation time.^38^^,^^73^ Eleven studies found a positive association between burnout and years of service,^29^^,^^38^^,^^41^^,^^44^^,^^48^^,^^54^^,^^57^^,^^63^^,^^75^^,^^80^^,^^81^^,^ whereas five found a negative association.^33^^,^^62^^,^^69^^,^^78^^,^^83^ The work-related consequences of burnout included a lack of job satisfaction,^24^^,^^33^^,^^45^^,^^55^ and an intention to change jobs.^24^^,^^34^^,^^43^^,^^47^ Burnout was also significantly associated with physical or psychological illness,^27^^,^^62^^,^^65^^,^^67^^,^^76^^,^^81^ smoking,^24^^,^^38^^,^^76^ a lack of exercise,^38^^,^^60^ and the distance travelled to work.^59^ The distance travelled to work and being asked to complete work tasks beyond the individual’s expertise were associated factors only in low-income and lower-middle-income countries. Protective factors identified included exercise, rest breaks and vacation time.^38^^,^^60^^,^^73^

### Quality assessment and publication bias

The risk of bias was calculated for each study: 46.7% of studies (28/60) scored between 5 and 7 points, which indicated a moderate risk of bias, and 53.3% (32/60) scored between 8 and 10 points, which indicated a low risk of bias. Studies scored well in domains relating to internal validity but less well in domains related to external validity, such as representative sampling frames and sampling methods. 

The Doi plot for a high depersonalization subscale score was symmetrical, with a low LFK index (0.03), which suggests a low risk of publication bias. However, the Doi plots for a high emotional exhaustion subscale score and a high personal accomplishment subscale score demonstrated minor asymmetry, with an LFK index of –1.08 and –1.11, respectively, which suggests a small risk of publication bias. Full details of the risk of bias assessment are available from the data repository.^17^

## Discussion

Our findings suggest that the prevalence of burnout among primary health-care professionals in low- and middle-income countries is substantial, perhaps unsurprisingly in view of the workforce and resource shortages in these countries.^14^^,^^84^ However, given that the consequences of burnout include increased sick leave, staff turnover and emigration, there are implications for workforce planning and the recruitment and retention of primary health-care professionals in countries where understaffing is already a critical issue. Any increased desire to emigrate could exacerbate the so-called brain drain from these countries to high-income countries.^8^ Policy-makers in low- and middle-income countries may need to work with policy-makers in high-income countries to identify solutions.

We found that the prevalence of emotional exhaustion and depersonalization was highest among primary care nurses, whereas the prevalence of reduced personal accomplishment was highest among CHWs. The high prevalence of burnout among nurses may affect patient safety as they are the main providers of community health care in some low- and middle-income countries. Longitudinal studies are needed to identify causal factors and to determine ways of reducing work demands on primary care nurses. One solution may be to increase the number of family physicians to provide professional support and clinical expertise. However, burnout is also common among family physicians and, therefore, any restructuring of roles and responsibilities must bear this in mind. Although international studies suggest that overall burnout levels among family physicians are similar in low- and middle-income countries and high-income countries, there are differences in the prevalence of each dimension of burnout for different cadres. For example, the prevalence of depersonalization is lower among primary care nurses in high-income countries than in low- and middle-income countries.^85^^,^^86^ This result may reflect differences in the responsibilities, workload and type of work expected of primary care nurses in low- and middle-income countries, where they are often responsible for diagnosis, treatment and performing basic procedures.^87^ Additionally, in contrast to observations in high-income countries,^24^ studies in our review suggest that reduced personal accomplishment is the most prevalent dimension of burnout for family physicians and CHWs in low- and middle-income countries. These results may reflect limited opportunities for further education, professional development and career progression in these countries. Policy-makers need to be aware of these differences, to work actively to identify individuals most at risk of burnout and to develop targeted interventions.

We were unable to compare findings from the three studies conducted during the COVID-19 pandemic with pooled pre-pandemic data because different measurement tools were used. However, the estimated overall prevalence of burnout in two of these studies was higher than the pooled prevalence we found for the individual Maslach Burnout Inventory subscales,^69^^,^^70^ which is in line with the findings of a global survey of health-care professionals that used a single-item scale to assess burnout during the COVID-19 pandemic and found a prevalence of 51%.^88^ Additionally, we found no clear difference in burnout prevalence between upper-middle-income countries and lower-middle-income and low-income countries. Again, this result was partly due to differences in the definition of burnout and in the measurement tools used, which made comparisons difficult.

In line with previous research,^89^ we found conflicting evidence on the association between burnout and sex. This outcome may have been due to: differences in how men and women experience burnout;^89^ cultural differences in sex roles;^71^ or cultural and sex differences in the importance of protective factors such as social support.^90^^,^^91^ Our findings suggest that burnout is more common in younger age groups. Younger professionals early in their careers may have greater family responsibilities, which could lead to increased conflict between work and home life and which, combined with lower professional self-efficacy, could result in a higher risk of burnout.^92^ In contrast to studies from high-income countries,^93^ 11 studies in our review found that the prevalence of burnout also increased with the number of years of service; it may be that limited opportunities for career development in low- and middle-income countries lead to frustration and burnout over the years. Our findings imply that burnout prevalence peaks in health workers both at an early career stage and much later in their careers. Consequently, policies and interventions to mitigate and prevent burnout should be targeted at these two career stages.

The evidence from our review confirms, as previously established,^93^ that burnout is associated with heavy workloads, few workplace resources, insufficient workplace support and conflict at work. One study conducted during the COVID-19 pandemic found that increased exposure to COVID-19 patients and the requirement to supply one’s own personal protective equipment were both positively associated with burnout.^68^ Another highlighted the need for specific pandemic training and increased organizational resources and support.^69^ These results are in line with findings from high-income countries, which highlight the increased workload and stress associated with exposure to COVID-19 patients, the need for extra training and support, and the importance of adequate personal protective equipment.^12^^,^^88^ Several studies in our review identified factors that protected against burnout, such as regular exercise, regular rest breaks and time away from work,^38^^,^^60^^,^^73^ which could be incorporated into the culture of primary care.

The geographical spread of studies in our review highlights the dearth of research on primary care burnout in low- and middle-income countries, specifically in Africa and South-East Asia, which are the WHO regions with the greatest shortages of health-care professionals.^84^ Moreover, most studies were performed in upper-middle-income countries, which limits the generalizability of our results to lower-resource settings. This finding highlights the urgent need for research in low-income and lower-middle-income countries. Importantly, 43% of studies in our review were published from 2019 onwards, possibly reflecting increasing awareness that a healthy primary care workforce is essential for achieving UHC.^13^

Study heterogeneity was high due to the breadth of primary health-care professionals included, the geographical spread of the studies and the variety of burnout measurement tools used. The variety of cultures, economies, disease burdens and political, educational and health systems in study countries would have resulted in differences in workload, resource availability and training, which may have contributed to large variations in the working environment and personal coping strategies between countries. However, the quality of the studies was good as no study was assessed as having a high risk of bias.

We conducted this study using a robust systematic review method and preregistered the study protocol on the PROSPERO website which ensured transparency. However, searches were limited to electronic databases and reference lists. Grey literature was not searched, which means that some data may have been missed, although the risk was small.^94^ Another limitation was the use of Google Translate rather than translators, which may have introduced errors at the data extraction stage. However, a recent study suggested that Google Translate is adequate for data extraction.^95^ One third of the studies retrieved by our searches and fulfilling our inclusion criteria were in languages other than English. Of the 25 studies from the Americas, 19 were not published in English. Excluding these studies would have excluded a considerable amount of regional data.

The findings of this review suggest that over half of primary health-care professionals in low- and middle-income countries have a moderate or high level of emotional exhaustion or reduced personal accomplishment and over a third have a moderate or high level of depersonalization. These results have implications for the health of the primary care workforce, staffing levels and the quality of care. It is necessary to identify protective factors against burnout, such as workplace support, continuing education and regular rest breaks, and to incorporate them into primary care. Further research should be conducted to provide better estimates of the prevalence of burnout and to explore its determinants, especially in underrepresented countries in Africa and South-East Asia, where workforce shortages are greatest. Additionally, this review highlighted the difficulty of making comparisons across regions, countries and professional groups when different measurement tools and definitions of burnout are used. There is, therefore, a need for an international consensus on a definition of burnout and on outcome measures to enable comparisons within burnout research.
